# Building the case for the use of gut feelings in cancer referrals: perspectives of patients referred to a non-specific symptoms pathway

**DOI:** 10.3399/BJGP.2021.0275

**Published:** 2021-11-30

**Authors:** Claire Friedemann Smith, Benedikte Møller Kristensen, Rikke Sand Andersen, Sue Ziebland, Brian D Nicholson

**Affiliations:** Nuffield Department of Primary Care Health Sciences, University of Oxford, Oxford, UK.; Research Unit for General Practice, Department of Public Health, Aarhus University, Aarhus, Denmark.; Department of Public Health, Research Unit of General Practice, University of Southern Denmark, Odense, Denmark.; Nuffield Department of Primary Care Health Sciences, University of Oxford, Oxford, UK.; Nuffield Department of Primary Care Health Sciences, University of Oxford, Oxford, UK.

**Keywords:** cancer, clinical decision making, primary health care, referral and consultation

## Abstract

**Background:**

Gut feelings may be useful when dealing with uncertainty, which is ubiquitous in primary care. Both patients and GPs experience this uncertainty but patients’ views on gut feelings in the consultation have not been explored.

**Aim:**

To explore patients’ perceptions of gut feelings in decision making, and to compare these perceptions with those of GPs.

**Design and setting:**

Qualitative interviews with 21 patients in Oxfordshire, UK.

**Method:**

Patients whose referral to a cancer pathway was based on their GP’s gut feeling were invited to participate. Semi-structured interviews were conducted from November 2019 to January 2020, face to face or over the telephone. Data were analysed with a thematic analysis and mind-mapping approach.

**Results:**

Some patients described experiencing gut feelings about their own health but often their willingness to share this with their GP was dependent on an established doctor–patient relationship. Patients expressed similar perspectives on the use of gut feelings in consultations to those reported by GPs. Patients saw GPs’ gut feelings as grounded in their experience and generalist expertise, and part of a process of evidence gathering. Patients suggested that GPs were justified in using gut feelings because of their role in arranging access to investigations, the difficult ‘grey area’ of presentations, and the time- and resource-limited nature of primary care. When GPs communicated that they had a gut feeling, some saw this as an indication that they were being taken seriously.

**Conclusion:**

Patients accepted that GPs use gut feelings to guide decision making. Future research on this topic should include more diverse samples and address the areas of concern shared by patients and GPs.

## INTRODUCTION

GPs’ gut feelings are defined as a sense of alarm or reassurance despite a lack of specific indications or certainty around the diagnosis.^1^ A systematic review recently described gut feelings as triggered by rapid summing up of verbal and non-verbal cues in the context of the GP’s clinical knowledge and experience.^2^ GPs have reported relying on gut feelings, particularly when caring for patients whose presentation falls into the ‘grey-area’ of primary care practice, where clinical guidance does not adequately address the patient’s presentation.^3^^,^^4^ In such cases, gut feeling served as the prompt for further clinical enquiry and investigation^2^^,^^3^ and may be a factor in the large proportion of UK GPs that said they would ignore guidance if they believed their patient needed cancer investigations.^5^

Research has shown that the presence of a GP’s gut feeling was associated with a greater proportion of patients being referred for investigations and increased the odds that cancer will be diagnosed.^2^ Despite gut feelings’ potential effect on the patient’s diagnostic journey, few studies have examined the role of gut feelings from the patient perspective. The small amount of available research has reported that patients’ concerns can trigger gut feelings in their GP,^6^ but that GPs’ willingness to respond to concerns is influenced by their opinion of their patient’s personality, with those regarded as ‘worriers’ less likely to have concerns noted than those considered ‘sensible’.^7^

As the authors have discussed previously,^3^^,^^8^ the utility of gut feelings may lie in helping to deal with the uncertainty inherent to primary care where there may be many alternative explanations for the patient’s symptoms. This uncertainty faces the GP trying to find the best route to care for patients who may have early-stage, undifferentiated illness, and the patient deciding whether to consult. Qualitative studies have found that patients report instinctively knowing that something is seriously wrong with their health and this influences their decision to seek help.^9^^,^^10^ GPs may find it difficult to share with their patients that clinical decisions are often made under conditions of uncertainty rather than by following objective indications, and GPs have voiced concerns about appearing ‘unscientific’ when using gut feelings.^3^^,^^11^^,^^12^ To the authors’ knowledge, no studies have explored whether patients share this concern.

Patients experiencing non-specific symptoms reportedly face a longer period before referral and diagnosis, with the lack of site-specific symptoms to guide referrals and investigations suggested as a reason for this.^13^ The aims of this study were to address the lack of the patient perspective in gut feeling research, to explore what patients who had been recently referred to a non-specific symptoms cancer pathway based on a gut feeling considered the appropriate role for gut feelings in clinical decision making, and to compare their views with those of GPs in the same study.^3^ The findings are presented following the Standards for Reporting Qualitative Research (SRQR) checklist.^14^

**Table 1:** How this fits in

Despite the reported diagnostic utility for cancer of GPs’ gut feelings and the role they may play in facilitating diagnosis through prompting investigation, research has not explored the use of gut feelings in clinical decision making with patients. This study found that patients were supportive of the use of gut feelings if they facilitated investigations but cautioned against their use if it meant that investigations would be deferred or denied. Patients discussed the difficulty facing GPs of having to fit individuals to referral ‘tick boxes’ in order to make a referral, were aware of the time-pressured and resource-limited conditions of primary care practice, and raised these as reasons for why GPs’ use of gut feelings is justified. Patients share GPs’ concerns around gut feelings overburdening NHS resources and increasing the risk of negligence and litigation, and these should be investigated.

## METHOD

### Recruitment

Patients were eligible to take part if they had been referred by their GP to a nonspecific cancer symptoms referral pathway operating in Oxfordshire, UK, that included ‘GP clinical suspicion of cancer or serious disease/GP gut feeling’ as a referral criterion.^15^ The pathway aims are to provide GPs with an urgent route to refer patients experiencing non-specific symptoms, and to provide patients with a diagnosis rather than simply ruling out cancer. Patients referred between October 2018 and October 2019, and agreeing to receive information about related research projects, were identified from the pathway database and contacted via a mail-out. None of the research team involved in the recruitment and conduct of the interviews were involved in the provision of care on the pathway, and patients was informed that their decision to participate (or not) would have no impact on their care. The recruitment packs included an introductory letter and participant information sheet explaining the study’s focus on gut feelings. Patients were requested to contact the study team should they have any questions or wish to take part.

### Sample

Interviews lasting on average 54 min (range 25–73 min) were conducted with 21 of the 22 patients who responded to the invitation to participate (5% response rate). One patient dropped out of the study for health reasons. The patients had been referred based on their GP’s gut feeling in combination with ≥1 of the other pathway referral criteria, all patients were white British, were aged between 47 and 90 years (mean 69 years), and 12 (57.1%) were female. The sample reflects the demographics of all patients referred to the non-specific symptoms pathway, where the mean age was 69 years (range 40–97 years) and 57.8% were female, and is similar to the demographics of the non-responders (mean age 70 years, 56.9% female). Three of the patients had received a cancer diagnosis through the pathway.

#### The interviews

Interviews were conducted by one of two experienced qualitative anthropologists, either face to face or over the telephone between November 2019 and January 2020. Telephone interviews were offered to facilitate recruitment of patients who may have been unable to travel or been unwilling to have a researcher in their home, and were conducted with five patients (24%). The semi-structured interview schedules were informed by the authors’ systematic review^2^ and a patient and public involvement (PPI) group. All interviews began with confirming consent, and the patients were given an opportunity to ask questions. If an interview was conducted over the telephone, the signed consent form was returned before the interview took place. Interviews began with the patient’s account of the circumstances surrounding their referral to the pathway before a discussion of the patient’s views on gut feelings as part of clinical decision making. Additionally, the patient interview schedules contained questions mirroring those in the GP interview schedules to allow comparison of responses. These primarily involved whether gut feeling was an acceptable referral criterion, how it should be used, and how it should be communicated (Supplementary Appendix S1).

### Analysis

The interviews were digitally recorded, transcribed verbatim, and anonymised. Patients were given the option to receive a copy of their anonymised transcript but were not asked to check the transcripts. The transcripts were coded by one author using NVivo (version 12) software into anticipated and emergent themes. The ‘one sheet of paper’ (OSOP) mind-mapping method^16^ and constant comparison were used to explore relationships between the interview extracts grouped within each theme during an analysis workshop attended by four of the authors. During the analysis it became clear that two of the themes from the interview study with GPs^3^ were also present in the patient interviews. These themes were: building a case for decisions based on gut feelings, and gut feelings and the GP’s professional role. These themes are presented below with commentary on how they relate to the matching theme in the GP interviews and the wider literature on gut feelings.

### Patient and public involvement

A focus group was held in November 2018 with five patients to gather feedback on the interest and relevance of GPs’ and patients’ gut feelings for cancer and other serious illness to patients, and on the draft interview schedule.

## RESULTS

### Patients’ own experiences of gut feelings

While the majority of each interview focused on GPs’ use of gut feelings, some patients also described their own experiences of gut feelings. These patients described a gut feeling that something was wrong, based on their knowledge of what was normal for them. When gut feelings were experienced, seeking medical help was described as a sensible reaction:

*‘It’s your body, you know if something is wrong, so you take it further. If you’re sensible that’s what you do.’*

(Participant [P]06, female [F])

For some patients, their relationship with their GP influenced whether they would mention their own gut feeling in the consultation. The sense that there was a shared understanding that they consulted judiciously (‘not every 5 minutes’) appeared to influence whether they would express their gut feelings:

*‘I think that* [GP trust in a patient’s gut feeling] *comes from knowing your patient doesn’t it?* […] *if you’ve got a patient who hasn’t been to see you for months or years and they’re saying something’s not quite right, well you know that they’re not somebody who comes every five minutes, do you know what I mean?’*

(P21, F)

### Building a case for decisions based on gut feelings

Similar to the authors’ discussions of gut feelings with GPs, the patients mentioned the nebulous nature of gut feelings, describing them as a sense that something was wrong for which it was hard to determine the origins. The legitimacy for the use of GP gut feelings in the consultation was seen as coming from a combination of clinical knowledge and use at the beginning of an investigative process:

*‘Yes, I think it’s* [using gut feelings] *a very good idea. I’m sure there are lots of signals, which are quite invisible, not, invisible is the wrong word but hard to define what it is you’ve picked up*. *’*

(P15, F)

*‘It’s* [gut feeling] *a certainty that they* [GPs] *know something is wrong and in relation to knowledge which they have.’*

(P22, male [M])

Descriptions of gut feeling prompting evidence gathering to provide objectivity to the suspicion were common. Patients described GPs as: *‘using it* [gut feeling] *in a scientific way’* (P2, F) or gut feeling *‘orientating’* (P13, M, received cancer diagnosis) GPs’ thoughts before further tests and investigations were carried out. While some patients were happy for gut feeling to be a criterion for referral and investigations in its own right, others suggested that legitimate gut-feelingbased referrals could only be made if they were supported by concerning symptoms. Additionally, some patients stated that decisions to delay or not investigate should not be based on gut feeling alone, and should not go against guidance:

*‘Oh I think if their patient is, showing symptoms* […] *because otherwise, would you be sending a patient unnecessarily and wasting loads of money?* […] *I do think it’s acceptable* [use of gut feelings] *, yes. In the right circumstances though*. *’*

(P19, F)

*‘I mean the gut feeling could be there’s something wrong* [or] *“Oh for God’s sake, there’s nothing wrong.”* […] *No, I think if you’re dealing with the public I don’t think you can do that* [take no action based on a gut feeling] […] *there’s a suing culture.’*

(P04, F)

*‘I think* [GPs] *should act on gut feeling, but* [GPs] *should also follow protocol.’*

(P10, F, received cancer diagnosis)

This contrasts with the definition of gut feelings that they may occur without demonstrable causes and highlights the difficulty in separating symptoms from gut feelings. It also echoes the concern expressed by GPs that gut-feeling-based referrals could increase unnecessary referrals.

As a way to judge how legitimate a particular GP’s use of gut feeling was, it was suggested that *‘the results of those* [gut feelings] *’* (P21, F) could be used to assess the proportion of patients for whom the GP had experienced a correct gut feeling:

*‘… for me, the important bit would be* […] *looking at it, and saying, “OK, how much of what I’ve done this week, this month, has been on, on gut, and what was the outcome of that?” And if they find that they’re one in ten right, then they’ve got a problem.’*

(P20, M)

Ultimately, for a number of patients the presence of concern in a GP, with training, skills, and experience, and the potential to rule out or diagnose serious disease earlier, provided legitimacy for the use of gut feelings. As such, the patients’ perspectives correspond closely to those of the GPs that we interviewed, who agreed that experience and knowledge are fundamentally important to reliable gut feelings:

*‘… because they may have seen people in that position before, and it may have started* […] *years before something more serious happened. So, it could be an indicator that although there’s nothing really nasty there now, there could be something there in the future. So I guess, you know it’s based on their experience, that they, they have that* [gut] *feeling, but they just can’t put a finger on it* . *‘*

(P18, M)

Empathy was also mentioned as a characteristic that was important to the development of gut feelings. Here again, the potential for gut feeling to appear ‘unscientific’ was reduced by emphasising that a good GP would use the ‘short cut’ that gut feeling offered but then ‘validate’ it:

*‘I think the intuitive or the gut feeling, the doctor who perhaps has that empathy* […] *can miss out at times some of the process.* […] *But equally I think* […] *even if they take the short cut, they’ll then, it might get to them to the answer sooner, but they’ll still validate it* . *’*

(P20, M)

The case for the use of gut feelings was also made by some patients by raising the challenges inherent in primary care. One patient reasoned that gut feeling was one of the *‘top’* diagnostic tools available to GPs, noting that GPs use them to navigate the often uncertain environment of primary care, where there is no time to mull over decisions, and little access to immediate testing:

*‘So I actually admire the GP that actually goes with gut feeling and I think that as a diagnostic tool, it comes pretty near the top because a GP is out there, without a safety net. He hasn’t got the backup facilities* […] *he’s got to make fairly quick decisions as to where to proceed. Maybe seven out of ten cases are straightforward, you know. You can deal with it. But there’s always this grey area where you’re a bit stuck.’*

(P04, F)

### Gut feelings and the GP’s professional role

In many of the conversations, the professional role of the GP was described as necessitating the use of gut feelings. For these patients, one of the primary roles of the GP was to enable access to investigations that could provide a diagnosis, and as such gut feelings facilitated the patient’s progression towards diagnosis:

*‘I should think a lot of doctors’ stuff is gut feeling isn’t it? And that’s why they send people for tests in hospitals, because they can’t, they can’t diagnose straightaway like that, so their gut feeling is you’ve got something, you know the patient may have something wrong with them, so you send them for a test.’*

(P05, M)

For a number of patients, the GP’s professional role to arrange access to other NHS services, and an appreciation for the challenge of the *‘grey area’* (P04, F) in general practice, made gut feelings a useful tool for GPs, more so than other specialties. The requirements of that role were also, however, described as a hindrance to the use of gut feelings. Barriers to the use of gut feelings included the requirement to refer patients to specific specialties and to ensure that a predetermined set of criteria were met. Patients stated that such requirements should not prevent investigation that the GP thought was necessary:

*‘*… *you’ve got to put them in the pigeonhole to send them to the hospital. And you have to go through these, excuse the word, bloody protocols* . *’*

(P04, F)

*‘I say that gut feeling comes with experience, from experience and knowledge* […] *I think we ought to take it seriously, you know, I hate to think that we get to a place where, you can’t be referred on unless you, you do all the tick boxes.’*

(P21, F)

GPs were often described by patients as having a broad knowledge base: *‘they learn a bit about everything, the general practitioner’* (P02, F), which is built on throughout their careers. Experience was described as fostering more expertise than could be gained through training alone, and influenced gut feelings directly so that, as experience increased, so too did their reliability:

*‘*… *even though you’ve been trained, you need the experience of being a doctor, and I should imagine you get better and better as you go along* […] *a doctor’s gut feelings get better as they go along* . *’*

(P05, M)

### Gut feelings and communicating concern

In view of GP’s expertise and their role in navigating undifferentiated and often early-stage illness, awareness that the GP was acting on a gut feeling communicated to the patient that they were being taken seriously. This may be particularly important to these patients who all presented with non-specific symptoms that can be difficult to *‘pigeonhole’*. (P04, F):

*‘She just said, “My gut feeling is there’s something not quite right here” and do you know what, that was such a relief* […] *I just felt I was being taken seriously* . *’*

(P21, F)

Some patients mentioned that they had known their GP for a number of years and they valued the relationship and trust. This continuity, which has been the traditional cornerstone of general practice, added credibility to the GP’s use of gut feelings:

*‘He did tell me that was his reasoning* [referral on a gut feeling for cancer] *. You know I’ve known him from, well about thirty years now, and he knows I’m not the type to be a dramatist or anything* […] *I would be quite happy to sort of think well, “Yes OK, I’ll get it checked out”.’*

(P07, F)

## DISCUSSION

### Summary

The aim of this study was to explore what patients thought about the role of gut feelings in clinical decision making in primary care and compare their views with those of the GPs who were spoken to in parallel interviews.^3^ Patients who described their own gut feelings said they resulted from knowing what was normal for their own bodies. The relationship between the GP and their patient was viewed as important for both the development of reliable gut feelings in the GP and the successful communication of concern in the consultation. On both counts, these corresponded to the views expressed in the GP interviews.

Patients recognised that GPs needed to differentiate between self-limiting and serious conditions in a clinical environment where time and access to investigations is limited. Like GPs, patients also raised the issue of the ‘grey area’ in primary care, where making the distinction between serious and non-serious illness was difficult and where gut feelings could provide the impetus for investigations. The belief that gut feelings were based on the clinical knowledge and experience of the GP was also shared by patients and GPs.

### Strengths and limitations

The main strength of this study is that it explores patient voices that have been largely absent from research into the use of gut feelings in primary care. All the included patients had been referred based on a gut feeling to a suspected cancer pathway and so were able to express their thoughts on the topic with reference to a recent, relevant example of gut feeling being used by a GP. The authors acknowledge, however, that because these patients had generally positive experiences of being referred on a gut feeling, this may have engendered greater support for the use of gut feelings than there would be in the general population.

A limitation is that all the patients were white British. While no research has examined how ethnicity may affect the expression or use of gut feelings for serious illness either from the perspective of the patient or the GP, a patient’s ethnicity has been associated with increased consultations, with patients of black, Asian, and mixed ethnicity having a greater chance of attending their GP ≥3 times before hospital referral than white patients.^17^ Other studies have suggested a connection between ethnic group and fatalism, and, for some groups, body vigilance.^18^^,^^19^ Furthermore, if the patient’s preferred language is not the same as the GP’s, difficulties in explaining symptoms or a gut feeling, which is difficult to describe even in the informant’s native language,^2^ may harm the development and use of gut feeling. As such, it is conceivable that ethnic background may influence the GP’s use of the ‘short cut’ to investigations that gut feelings provide, the patients’ experience of being taken seriously by their GP, and their views on gut feelings, but this is beyond the scope of this study. Finally, the use of telephone interviews may be seen as a limitation. Telephone interviews are sometimes said to be lower quality because they are generally briefer and the lack of visual cues can harm the development of rapport.^20^ Any difference in quality, however, is not clear-cut, with advantages and disadvantages in each modality.^21^ Reports of high-quality, rich data gathered through telephone interviews are also common in the literature.^22^ The interviews described here were conducted according to patient preference and by very experienced researchers.

### Comparison with existing literature

Patients’ descriptions of the triggers of gut feelings as departures from usual patterns of health that are facilitated by familiarity with what constitutes ‘normal’ have been described by GPs previously.^2^^,^^3^ Patients also described changes from ‘normal’ as triggers for their own gut feelings and explained that this change could include the experience of a symptom. The separation of symptom recognition from GP gut feeling is notoriously difficult, but it is thought that GPs’ gut feelings are based on complex pattern recognition and clinical experience rather than symptom recognition alone.^2^ Further research should examine whether the triggers of patients’ gut feelings differ from the triggers of GPs’ gut feelings.

GPs are reported to support the use of gut feelings as a prompt for further investigations and evidence gathering,^3^^,^^7^^,^^23^ and this study adds patient support as well. The patients in the present study agreed with reports that GPs believe that greater clinical experience leads to more reliable gut feelings,^2^^,^^3^^,^^24^ and that continuity of care and GP characteristics such as empathy facilitate the development of gut feelings.^3^ The findings of the authors’ previous related studies suggest gut feelings are triggered by complex pattern recognition facilitated by knowledge of the patient, clinical experience, and empathy.^2^^,^^3^ Such a definition would fit with research into fast and slow thinking as part of clinical reasoning where increasing experience moves reasoning away from hypothetico-deductivism towards pattern matching.^25^^–^28 Additionally, increasing understanding of gut feelings, GPs’ efforts to distance gut feelings from the unscientific, and the statement from one of the patient interviewees that the term is *‘jargon’* (P22, M) suggest that it may be time to discuss them as clinical intuition rather than gut feeling.^29^

When the potential drawbacks of using gut feelings as a part of clinical decision making in primary care were discussed, GPs and patients shared concerns that requesting investigations or making referrals on gut feelings could overburden NHS resources.^3^ While GPs also mentioned a desire to avoid over-investigating,^3^ this concern was not mentioned by patients. It has been previously reported that patients have a strong preference for testing even at low levels of cancer risk,^30^ will tolerate high levels of false positive findings at screening,^31^ and generally have limited awareness of overdiagnosis.^32^^,^^33^ It has also been reported that the importance placed on finding cancer quickly and treating it without delay often outweighs considerations of potential harms caused by investigation.^32^ These factors may explain why patients do not raise concerns about the potential harms of over-investigation.

Despite their support for GPs’ gut feelings to prompt investigations, the patients did not support gut feeling being used in decisions to delay or not investigate a patient. Instead, patients said that GPs should follow guidance over their gut feeling if guidance recommended investigations, and raised the possibility of complaints if symptoms were not investigated, a concern that GPs have also raised.^3^ The authors suggest that patients’ statements that gut feeling is sufficient to justify referral but not to deny it likely reflects their understanding that cancer should be diagnosed without delay, rather than the reliability or usefulness of either as a decision-making tool.

### Implications for research and practice

While the patients in this study expressed very few concerns over GPs’ use of gut feelings, they agreed with GPs’ concerns over the additional pressure they may put on resources for investigation or specialist review, as well as complaints or legal repercussions if gut feelings of reassurance were relied on to delay or avoid a referral.^3^ The actual likelihood of these concerns should be assessed. Stolper and colleagues previously examined how gut feelings are dealt with at Dutch disciplinary tribunals.^34^ That study is now a decade old, and, despite the enduring concern that gut feelings could lead to negligence and litigation, no further research has sought to establish its legitimacy. The authors suggest this as an area for future research.

A secondary analysis of the English national GP Patient Survey found that patients value being taken seriously by their GP and that this was the factor most strongly associated with patients’ confidence and trust in their GP.^35^ Similarly, the participants in the current study likened the presence of a gut feeling of alarm in their GP to being taken seriously, and this could engender trust and satisfaction with the consultation. The experiences of participants in the present study with GP gut feelings was, however, generally positive and this may have influenced their willingness to support their use. The relationship between satisfaction with consultations involving gut feeling and the perception of GPs’ gut feeling should be explored further and with broader samples, including ethnic minorities.

When used as a prompt for information gathering, the use of gut feelings appears to be acceptable to GPs and patients alike. This is contingent, however, on the GP prioritising symptoms and guidance, especially if the gut feeling suggests investigation is not necessary. Given the acceptance in principle of the use of gut feelings in primary care, efforts should be made to investigate concerns shared by GPs and patients over the pressure on resources and potential for complaints. Consistent, explicit recording of gut feelings will facilitate this.
